# Supplementary material for: Presenting decision-relevant numerical information to Dutch women aged 50–70 with varying levels of health literacy: Case example of adjuvant systemic therapy for breast cancer
Source: PLoS One. 2024 Sep 3;19(9):e0309668. doi: 10.1371/journal.pone.0309668 (PMC11371237; doi:10.1371/journal.pone.0309668)
Supplement: S1 File — (PDF) [file pone.0309668.s001.pdf]

Supplemental Material 1 - Side-effects formats

Possible side effects of hormone therapy

- Dry vagina and less sex drive
- Mood swings
- A clot in the blood vessels resulting in thrombosis or stroke
- Hot flashes and sweating
- Headache
- Pain in muscles, bones and/or joints

Possible side effects of chemotherapy

- Sore mouth and throat, faster nosebleed
- Deficiency of immune cells. This can cause you to develop a fever and become seriously ill.
- Anemia. This makes you feel tired and dizzy, and your concentration decreases.
- Prolonged fatigue
- Diarrhea or constipation, nausea, and vomiting
- Hair loss
- An altered sensation in the hands and feet, such as tingling or numbness.
- Flulike feeling and muscle strain

Format A. No probability information

Possible side effects of hormone therapy

Between 1 and 10 in 100 women who receive hormone therapy, experience:

- Dry vagina and less sex drive
- Mood swings
- A clot in the blood vessels resulting in thrombosis or stroke

More than 10 in 100 women who receive hormone therapy, experience:

- Hot flashes and sweating
- Headache
- Pain in muscles, bones and/or joints

Possible side effects of chemotherapy

Between 1 and 10 in 100 women who receive chemotherapy, experience:

- Sore mouth and throat, faster nosebleed
- Deficiency of immune cells. This can cause you to develop a fever and become seriously ill.
- Anemia. This makes you feel tired and dizzy, and your concentration decreases.

More than 10 in 100 women who receive chemotherapy, experience:

- Prolonged fatigue
- Diarrhea or constipation, nausea, and vomiting
- Hair loss
- An altered sensation in the hands and feet, such as tingling or numbness.
- Flulike feeling and muscle strain

Format B. Probability information in numbers without description

Possible side effects of hormone therapy

Between 1 and 10 in 100 women who receive hormone therapy, experience:

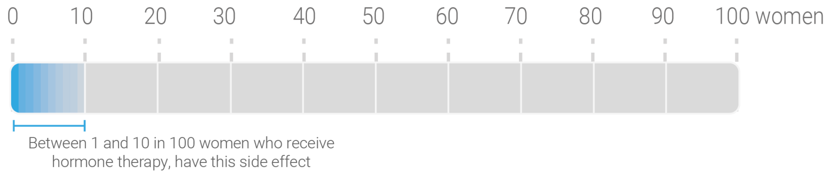

- Dry vagina and less sex drive
- Mood swings
- A clot in the blood vessels resulting in thrombosis or stroke

More than 10 in 100 women who receive hormone therapy, experience:

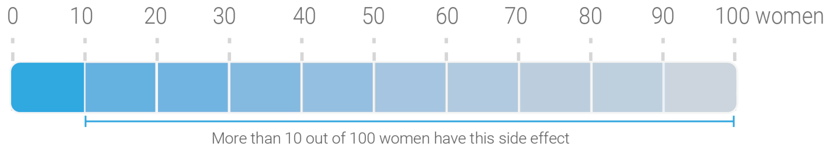

- Hot flashes and sweating
- Headache
- Pain in muscles, bones and/or joints

Possible side effects of chemotherapy

Between 1 and 10 in 100 women who receive chemotherapy, experience:

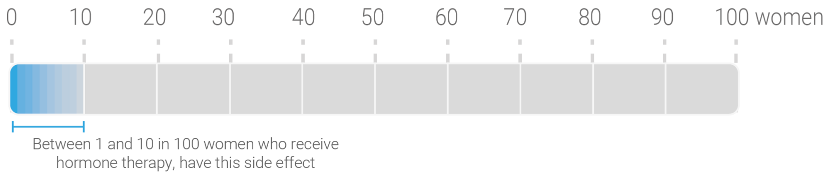

- Sore mouth and throat, faster nosebleed
- Deficiency of immune cells. This can cause you to develop a fever and become seriously ill.
- Anemia. This makes you feel tired and dizzy, and your concentration decreases.

More than 10 in 100 women who receive chemotherapy, experience:

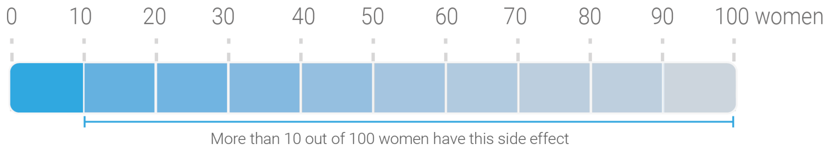

- Prolonged fatigue
- Diarrhea or constipation, nausea, and vomiting
- Hair loss
- An altered sensation in the hands and feet, such as tingling or numbness.
- Flulike feeling and muscle strain

Format C. Visualised probability information without description

Possible side effects of hormone therapy

Between 1 and 10 in 100 women who receive hormone therapy experience:

Dry vagina and less sex drive

The treatment makes the mucous membranes drier and thinner. This makes the vagina more sensitive to irritations. Complaints that you may suffer from: dryness, discharge, itching, blood loss, and pain during sex.

Is there anything that can be done about this side effect? No **Yes**

You can use a vaginal gel to reduce the pain during intercourse. Always discuss this with your attending physician

Does the side effect disappear after the treatment? No **Yes**

Mood swings

The treatment can cause or intensify feelings of depression because the treatment causes disturbances in the metabolism of the brain.

Is there anything that can be done about this side effect? No **Yes**

Talking to a healthcare provider or psychologist can help. Medicines can also be given against depressive feelings. In consultation with your attending physician, it may be decided to adjust or stop the hormone therapy.

Does the side effect disappear after the treatment? No **Yes**

A clot in the blood vessels resulting in thrombosis or stroke

Is there anything that can be done about this side effect? No **Yes**

If you experience this side effect, you may be given blood thinners. In consultation with your attending physician, it can also be decided to adjust or stop the hormone therapy.

Does the side effect disappear after the treatment? No **Yes**

Possible side effects of hormone therapy

More than 10 out of 100 women who receive hormone therapy experience:

Hot flashes and sweating

The treatments can make menopausal symptoms such as hot flashes and sweating come back or worsen.

Is there anything that can be done about this side effect? No **Yes**

Medicines can be given for hot flashes. It can also help not to eat or drink some things, such as coffee and spicy herbs.

Does the side effect disappear after the treatment? No **Yes**

Headache

You may experience headaches as a result of the treatment. You may also be sensitive to stimuli such as light and sound.

Is there anything that can be done about this side effect? No **Yes**

Painkillers may be given.

Does the side effect disappear after the treatment? No **Yes**

Pain in muscles, bones and/or joints

Treatment can make your bones more brittle, making them more likely to break.

Is there anything that can be done about this side effect? No **Yes**

The advice is to take calcium vitamin D tablets. The strength of your bones is checked every 2 to 3 years. If your bones become more brittle, bone-strengthening drugs may be given.

Does the side effect disappear after the treatment? No **Yes**

The advice is to continue taking calcium vitamin D tablets.

Possible side effects of chemotherapy

Between 1 and 10 in 100 women who receive chemotherapy experience:

Sore mouth and throat, faster nosebleed

Possible symptoms include: a dry or painful feeling in and around the mouth; sores on the gums, palate, tongue, cheeks, and lips; sensitivity to the temperature of food and drink; change or loss of taste; bleeding gums quickly; a nosebleed that stays longer.

Is there anything that can be done about this side effect? **No** Yes

Does the side effect disappear after the treatment? No **Yes**

Deficiency of immune cells. This can cause you to develop a fever and become seriously ill

Chemotherapy temporarily produces too few new blood cells. Fewer white blood cells can lead to reduced resistance and greater susceptibility to infections. An infection can be recognized by a fever. Sometimes you have chills.

Is there anything that can be done about this side effect? **No** Yes

Always contact your attending physician immediately. Antibiotics can then be given on time. This prevents you from becoming seriously ill due to an infection.

Does the side effect disappear after the treatment? No **Yes**

Anemia. This makes you feel tired and dizzy, and your concentration decreases

Chemotherapy reduces the production of new blood cells. Anemia is caused by a low red blood cell count. With anemia, you may suffer from: paleness, dizziness, palpitations, shortness of breath, worsening fatigue, and seeing black spots.

Is there anything that can be done about this side effect? No **Yes**

In severe complaints, a blood transfusion can be given.

Does the side effect disappear after the treatment? No **Yes**

Possible side effects of chemotherapy

More than 10 out of 100 women who receive chemotherapy experience:

Prolonged fatigue

You may feel exhausted all the time. Physical exertion may require more effort. You may also suffer from loss of concertation and memory problems. The most common complaints are: lack of energy, listlessness, less interest in the environment, and irritability

Is there anything that can be done about this side effect? **No** Yes

It is recommended to keep moving.

Does the side effect disappear after the treatment? **No** **Yes**

Some of the women have to deal with chronic fatigue. For some women, the energy returns.

Diarrhea or constipation, nausea, and vomiting

Diarrhea is caused by irritation of the mucous membrane of the intestine. You may experience: abdominal pain and cramps, and a frequent urge to go to the toilet. You may also experience retching and vomiting, little or no appetite, and stomach upset, such as feeling full or in pain.

Is there anything that can be done about this side effect? No **Yes**

Medicines can be given for nausea. It can also help to eat several small portions and not to eat or drink some things, such as coffee and spicy herbs.

Does the side effect disappear after the treatment? No **Yes**

Hair loss

Chemotherapy makes fewer or no new hairs. The hair becomes thinner, breaks quickly, or falls out. Usually, the hair on the head falls out first, but other body hair can also fall out.

Is there anything that can be done about this side effect? No **Yes**

Sometimes scalp cooling is possible to prevent hair loss.

Does the side effect disappear after the treatment? No **Yes**

An altered sensation in the hands and feet, such as tingling or numbness

You may develop the following complaints of hands and feet: itching, redness, pain or sensitivity, swelling, flaking or peeling of the skin, blisters, and infections of the skin and/or nails.

Is there anything that can be done about this side effect? **No** Yes

Does the side effect disappear after the treatment? **No** Yes

Flulike feeling and muscle strain

You may experience: a feeling of general discomfort (general malaise), headache, fever, muscle strain and pain in the bones, and loss of appetite.

Is there anything that can be done about this side effect? **No** Yes

Does the side effect disappear after the treatment? No **Yes**

Format D. Probability information in numbers with accompanying description

Possible side effects of hormone therapy

Between 1 and 10 in 100 women who receive hormone therapy experience:

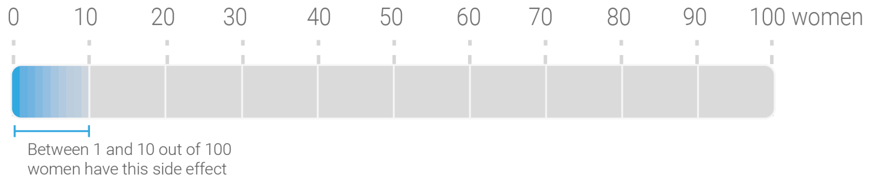

Dry vagina and less sex drive

The treatment makes the mucous membranes drier and thinner. This makes the vagina more sensitive to irritations. Complaints that you may suffer from: dryness, discharge, itching, blood loss, and pain during sex.

Is there anything that can be done about this side effect? No **Yes**

You can use a vaginal gel to reduce the pain during intercourse. Always discuss this with your attending physician

Does the side effect disappear after the treatment? No **Yes**

Mood swings

The treatment can cause or intensify feelings of depression because the treatment causes disturbances in the metabolism of the brain.

Is there anything that can be done about this side effect? No **Yes**

Talking to a healthcare provider or psychologist can help. Medicines can also be given against depressive feelings. In consultation with your attending physician, it may be decided to adjust or stop the hormone therapy.

Does the side effect disappear after the treatment? No **Yes**

A clot in the blood vessels resulting in thrombosis or stroke

Is there anything that can be done about this side effect? No **Yes**

If you experience this side effect, you may be given blood thinners. In consultation with your attending physician, it can also be decided to adjust or stop the hormone therapy.

Does the side effect disappear after the treatment? No **Yes**

Possible side effects of hormone therapy

More than 10 out of 100 women who receive hormone therapy experience:

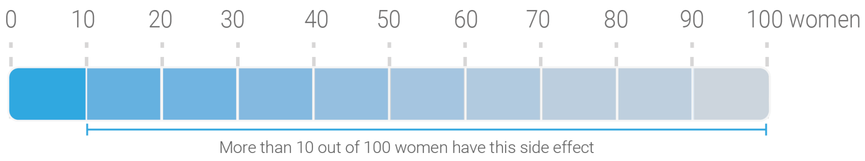

Hot flashes and sweating

The treatments can make menopausal symptoms such as hot flashes and sweating come back or worsen.

Is there anything that can be done about this side effect? No **Yes**

Medicines can be given for hot flashes. It can also help not to eat or drink some things, such as coffee and spicy herbs.

Does the side effect disappear after the treatment? No **Yes**

Headache

You may experience headaches as a result of the treatment. You may also be sensitive to stimuli such as light and sound.

Is there anything that can be done about this side effect? No **Yes**

Painkillers may be given.

Does the side effect disappear after the treatment? No **Yes**

Pain in muscles, bones and/or joints

Treatment can make your bones more brittle, making them more likely to break.

Is there anything that can be done about this side effect? No **Yes**

The advice is to take calcium vitamin D tablets. The strength of your bones is checked every 2 to 3 years. If your bones become more brittle, bone-strengthening drugs may be given.

Does the side effect disappear after the treatment? No **Yes**

The advice is to continue taking calcium vitamin D tablets.

Possible side effects of chemotherapy

Between 1 and 10 in 100 women who receive chemotherapy experience:

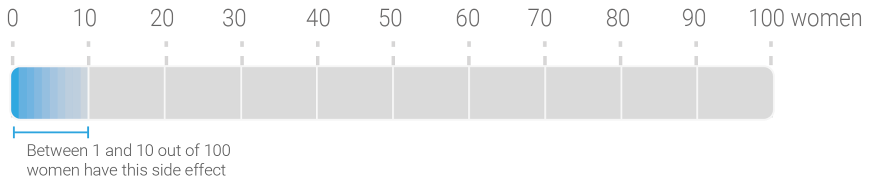

Sore mouth and throat, faster nosebleed

Possible symptoms include: a dry or painful feeling in and around the mouth; sores on the gums, palate, tongue, cheeks, and lips; sensitivity to the temperature of food and drink; change or loss of taste; bleeding gums quickly; a nosebleed that stays longer.

Is there anything that can be done about this side effect? **No** Yes

Does the side effect disappear after the treatment? No **Yes**

Deficiency of immune cells. This can cause you to develop a fever and become seriously ill

Chemotherapy temporarily produces too few new blood cells. Fewer white blood cells can lead to reduced resistance and greater susceptibility to infections. An infection can be recognized by a fever. Sometimes you have chills.

Is there anything that can be done about this side effect? **No** Yes

Always contact your attending physician immediately. Antibiotics can then be given on time. This prevents you from becoming seriously ill due to an infection.

Does the side effect disappear after the treatment? No **Yes**

Anemia. This makes you feel tired and dizzy, and your concentration decreases

Chemotherapy reduces the production of new blood cells. Anemia is caused by a low red blood cell count. With anemia, you may suffer from: paleness, dizziness, palpitations, shortness of breath, worsening fatigue, and seeing black spots.

Is there anything that can be done about this side effect? No **Yes**

In severe complaints, a blood transfusion can be given.

Does the side effect disappear after the treatment? No **Yes**

Possible side effects of chemotherapy

More than 10 out of 100 women who receive chemotherapy experience:

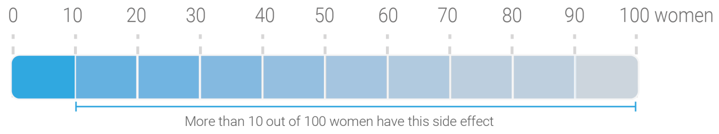

Prolonged fatigue

You may feel exhausted all the time. Physical exertion may require more effort. You may also suffer from loss of concertation and memory problems. The most common complaints are: lack of energy, listlessness, less interest in the environment, and irritability

Is there anything that can be done about this side effect? No Yes

It is recommended to keep moving.

Does the side effect disappear after the treatment? No Yes

Some of the women have to deal with chronic fatigue. For some women, the energy returns.

Diarrhea or constipation, nausea, and vomiting

Diarrhea is caused by irritation of the mucous membrane of the intestine. You may experience: abdominal pain and cramps, and a frequent urge to go to the toilet. You may also experience retching and vomiting, little or no appetite, and stomach upset, such as feeling full or in pain.

Is there anything that can be done about this side effect? No Yes

Medicines can be given for nausea. It can also help to eat several small portions and not to eat or drink some things, such as coffee and spicy herbs.

Does the side effect disappear after the treatment? No Yes

Hair loss

Chemotherapy makes fewer or no new hairs. The hair becomes thinner, breaks quickly, or falls out. Usually, the hair on the head falls out first, but other body hair can also fall out.

Is there anything that can be done about this side effect? No Yes

Sometimes scalp cooling is possible to prevent hair loss.

Does the side effect disappear after the treatment? No Yes

An altered sensation in the hands and feet, such as tingling or numbness

You may develop the following complaints of hands and feet: itching, redness, pain or sensitivity, swelling, flaking or peeling of the skin, blisters, and infections of the skin and/or nails.

Is there anything that can be done about this side effect? No Yes

Does the side effect disappear after the treatment? No Yes

Flulike feeling and muscle strain

You may experience: a feeling of general discomfort (general malaise), headache, fever, muscle strain and pain in the bones, and loss of appetite.

Is there anything that can be done about this side effect? No Yes

Does the side effect disappear after the treatment? No Yes

Format E. Visualised probability information with accompanying description
