# Supplementary material for: Presenting decision-relevant numerical information to Dutch women aged 50–70 with varying levels of health literacy: Case example of adjuvant systemic therapy for breast cancer
Source: PLoS One. 2024 Sep 3;19(9):e0309668. doi: 10.1371/journal.pone.0309668 (PMC11371237; doi:10.1371/journal.pone.0309668)
Supplement: S2 File — (PDF) [file pone.0309668.s002.pdf]

Supplemental Material 2 - Secondary outcome measures

Outcome measures	Items	Scale / response categories	Scale's Cronbach's alpha
Affect	Below are some words that describe different feelings and emotions. We want to know how you are feeling after reading the information. Please read each word and indicate to what extent you feel this way.<br>• Alert (PA)[a]<br>• Excited (PA)<br>• Enthusiastic (PA)<br>• Nervous (NA)[b]<br>• Inspired (PA)<br>• Distressed (NA)<br>• Determined (PA)<br>• Upset (NA)<br>• Scared (NA)<br>• Afraid (NA)<br>• Overwhelmed[c]<br>• Worried[c]	1 (very slightly or not at all) – 5 (extremely)	PA<br>.756 [exp 1]<br>.693 [exp 2]<br><br>NA<br>.940 [exp 1]<br>.952 [exp 2]
Hypothetical decision	• Suppose that you got this information from your doctor and that you now would need to make a choice about adjuvant treatment. What would you choose?	No additional treatment (option 1) / Hormone treatment (option 2) / Hormone treatment and chemotherapy (option 3)	
Decision confidence	• About this decision, I am currently	1 (not confident at all) – 10 (very confident)	
Decision uncertainty [Experiment 2 only]	• I am clear about the best choice for me<br>• I feel sure about what to choose<br>• This decision is easy for me to make	1 (strongly agree) –<br>5 (strongly disagree)	.863
Preparedness for decision-making [Experiment 2 only]	Did this information:<br>• Help you recognise that a decision needs to be made?<br>• Prepare you to make a better decision?<br>• Help you think about the pros and cons of each option?<br>• Help you think which pros and cons are most important?<br>• Help you know that the decision depends on what matters most to you?<br>• Help you organise your own thoughts about the decision?	1 (not at all) – 5 (a great deal)	.895
Perception of treatment effect [Experiment 1 only]	• Taking hormone treatment (option 2) reduces my chance of dying compared to taking no additional treatment (option 1)	1 (adding hormone treatment would not reduce the chance at all) – 10 (adding hormone treatment would reduce the chance a great deal)	

	• Taking hormone treatment and chemotherapy (option 3) reduces my chance of dying compared to taking only hormone treatment (option 2)	1 (adding chemotherapy would not reduce the chance at all) – 10 (adding chemotherapy would reduce the chance a great deal)	
Risk perception Hormone therapy [Experiment 2 only]	• The side-effects of hormone treatment seem to me:<br>• The side-effects of hormone treatment seem to me:<br>• The side-effects of hormone treatment seem to me:<br>• If I take hormone treatment, the chance that I will experience one of the side-effects is:<br>• I think I will experience one or more of the side-effects if I take the hormone treatment.<br>• Concerning the chance of getting one or more of the side-effects of hormone treatment, I am:	1 (not serious at all) – 10 (very serious)<br>1 (not at all dangerous to health) – 10 (very dangerous to health)<br>1 (without consequences for daily life) – 10 (with very significant consequences for daily life)<br>1 (very small) – 10 (very high)<br>1 (totally disagree) – 10 (totally agree)<br>1 (not worried at all) – 10 (very worried)	.874
Risk perception Chemotherapy [Experiment 2 only]	• The side-effects of chemotherapy seem to me:<br>• The side-effects of chemotherapy seem to me:<br>• The side-effects of chemotherapy seem to me:<br>• If I take chemotherapy, the chance that I will experience one of the side-effects is:<br>• I think I will experience one or more of the side-effects if I take chemotherapy.<br>• Concerning the chance of getting one or more of the side-effects of chemotherapy, I am:	1 (not serious at all) – 10 (very serious)<br>1 (not at all dangerous to health) – 10 (very dangerous to health)<br>1 (without consequences for daily life) – 10 (with very significant consequences for daily life)<br>1 (very small) – 10 (very high)<br>1 (totally disagree) – 10 (totally agree)<br>1 (not worried at all) – 10 (very worried)	.911
Evaluation of information	• The information is helpful when I have to make a decision about adjuvant treatment of breast cancer.<br>• I would recommend this information to other women with breast cancer.<br>• The information is clear.	1 (totally disagree) - 10 (totally agree)	.913 [exp 1]<br>.901 [exp 2]

Notes. [a]NA = Negative Affect. [b]PA = Positive Affect. [c]Worried and Overwhelmed were added to the original PANAS items.
