# Supplementary material for: Presenting decision-relevant numerical information to Dutch women aged 50–70 with varying levels of health literacy: Case example of adjuvant systemic therapy for breast cancer
Source: PLoS One. 2024 Sep 3;19(9):e0309668. doi: 10.1371/journal.pone.0309668 (PMC11371237; doi:10.1371/journal.pone.0309668)
Supplement: S3 File — (PDF) [file pone.0309668.s003.pdf]

Supplemental Material 3 - Numeracy and Graph Literacy

Methods

Measures

Numeracy was assessed using the three mathematical questions of Schwartz et al. [1] about probabilities and ratio concepts. The questions are 'Imagine that we flip a fair coin 1,000 times. What is your best guess about how many times the coin would come up heads in 1,000 flips?', 'In the Big Bucks Lottery, the chance of winning a $10 prize is 1%. What is your best guess about how many people would win a $10 prize if 1000 people each buy a single ticket to Big Bucks?', and 'In ACME Publishing Sweepsakes, the chance of winning a car is 1 in 1,000. What percent of tickets to ACME Publishing Sweepsakes win a car?'. The open-ended questions were coded as 1 (correct) or 0 (incorrect). A median-split was used for comparison between low (<2) and high (≥2) numeracy.

Graph Literacy was measured using the Short Graph Literacy (SGL) scale [2]. This scale consists of two open-ended questions and two multiple-choice questions regarding different graphical displays. Answers were coded as 1 (correct) or 0 (incorrect). A median-split was used for comparison between low (≤2) and high (>2) GL.

Data analysis

As with the analyses with health literacy (HL), the analyses with comprehension as an outcome variable were performed using cumulative odds ordinal logistic regression with proportional odds and the effect of format and numeracy/GL on the hypothetical decision was analysed using chi-square tests of association. The other outcome measures (Experiment 1: experienced affect, perception of treatment effect, decision certainty, evaluation of the information, realism check; Experiment 2: feeling informed, experienced affect, uncertainty, risk perception, preparedness for decision-making, decision certainty, evaluation of the information, realism check) were analysed using two-way ANOVAs, with format and numeracy/GL as independent variables.

Results: Experiment 1 – presentation of survival rates

Numeracy

In the first experiment, the first numeracy question was answered correctly by 66.7% of the total of 219 participants. The second question by 73.1% and the third question by 38.4%.

Numeracy – primary outcomes

There was no significant interaction between format and numeracy on gist comprehension, Wald $\chi^2(2) = 1.48$, $p = .477$. We found a significant main effect of numeracy on gist comprehension, Wald $\chi^2(1) = 4.22$, $p = .040$. This effect showed that women with high numeracy exhibited higher gist comprehension compared to women with low numeracy, with the odds of high-numerate women exhibiting higher gist comprehension being 2.11 (95% CI, 1.04 to 4.31) times that of low-numerate women.

Regarding the effects of format and numeracy on verbatim comprehension, a multinomial logistic regression was conducted[1], which showed no significant interaction $\chi^2(8) = 7.86$, $p = .447$. A multinomial logistic regression without interaction[2] was conducted, which showed no significant effect of format on verbatim comprehension, $\chi^2(8) = 12.54$, $p = .129$, but for numeracy, there was a statistically significant main effect, $\chi^2(4) = 20.50$, $p < .001$. This effect indicated that it was more likely for women to have 0 or only 1 verbatim question answered correctly if they have low numeracy compared to those with high numeracy.

Numeracy – secondary outcomes

Regarding experienced affect, there was a statistically significant interaction between numeracy and format for being worried, $F(2, 213) = 4.03$, $p = .019$, partial $\eta^2 = .04$. Therefore, an analysis of simple main effects was performed. Among those provided with the text format, there

[1] The model with interaction violated the assumption of proportional odds as assessed by a full likelihood ratio test, $\chi^2(15) = 54.807$, $p < .001$.
[2] The model without interaction also violated the assumption of proportional odds $\chi^2(9) = 58.366$, $p < .001$.

was a difference in being worried between women with low numeracy and women with high numeracy, F (1,213) = 7.18, p = .008 partial η^2 = .03, with women with high numeracy feeling more worried (2.98 ± 1.20) than women with low numeracy (2.26 ± 1.20), a mean difference of .72 (95% CI, .19 to 1.25). Other effects were not significant and neither were interactions or main effects for the other experienced affect outcomes (Positive Affect, Negative Affect, and overwhelmed).

There was a main effect for numeracy on the evaluation scores, $F(1, 213) = 6.97$, $p = .009$, partial η^2 = .03. Women with high numeracy evaluated the information more positively (marginal means 7.74 ± .16) than women with low numeracy (marginal means 7.08 ± .20), a mean difference of .66 (95% CI, .17 to 1.16).

Regarding perception of treatment effect of hormone therapy and the combination of hormone therapy/chemotherapy and decision certainty, there were no significant interactions or main effects. Similarly, for the hypothetical treatment decision (no additional treatment, hormone therapy, combination of hormone therapy/chemotherapy) there were no significant effects of numeracy.

However, there was a main effect for numeracy on the realism scores $F(1, 213) = 8.21$, $p = .005$, partial η^2 = .04. Women with high numeracy (marginal means 7.61 ± .16) were more likely to empathise with the hypothetical scenario than women with low numeracy (marginal means 6.89 ± .20), a mean difference of .72 (95% CI, .23 to 1.22).

Graph Literacy – primary outcomes

There was no significant interaction between format and GL on gist comprehension, Wald $\chi^2(2) = .08$, $p = .962$. We did find a significant main effect of GL, Wald $\chi^2(1) = 7.20$, $p = .007$. This effect showed that women with high GL exhibited better gist comprehension than women with low GL; the odds of higher GL women exhibiting higher gist comprehension was 3.00 (95% CI, 1.34 to 6.69) times that of lower GL women. Regarding verbatim comprehension, there was no significant interaction nor significant main effects.

Graph Literacy – secondary outcomes

Regarding experienced affect, there was a statistically significant interaction between GL and format on Positive Affect (PANAS PA), $F(2, 213) = 3.44$, $p = .034$, partial $\eta^2 = .03$. However, follow-up analysis showed no statistically significant differences. Interactions or main effects for the other experienced affect outcomes (Negative Affect, overwhelmed, and worried) were not significant.

Regarding perception of treatment effect of hormone therapy, there was a significant interaction between GL and format $F(2, 213) = 3.35$, $p = .037$, partial $\eta^2 = .03$. Therefore, an analysis of simple main effects was performed. Among those provided with the bar graph format, there was a difference in perception of treatment effect between women with low GL and women with high GL, F (1,213) = 5.54, p = .02 partial $\eta^2 = .025$, with lower GL women perceiving treatment effect as higher (7.04 ± 1.99) than higher GL women (5.96 ± 1.63). Other differences were not significant, and neither were the interactions or main effects for perception of treatment effect of the combination of hormone therapy/chemotherapy. Similarly, for evaluation of information, realism check, and decision certainty there were no significant interactions or main effects. The effect of GL on the hypothetical decision was also not significant.

Correlations between Health Literacy, numeracy and Graph Literacy

A chi-square test for association was conducted between HL and numeracy and between HL and Graph Literacy. There was a statistically significant association between HL and numeracy $\chi^2(1) = 3.85$, p = .050; φ = -1.33, p = .050. There was no statistically significant association between HL and Graph Literacy $\chi^2(1) = 1.38$, p = .240; φ = -.08, p = .240.

Results: Experiment 2 – side-effects information in addition to survival rates

Numeracy

In the second experiment, the first numeracy question was answered correctly by 63.1% of the total of 282 participants. The second question by 70.2% and the third question by 39.7%.

Numeracy – primary outcomes

There was no significant interaction between format and numeracy on gist comprehension of the trade-off, Wald $\chi^2(4) = 3.47$, $p = .483$. We did find a significant main effect of numeracy, Wald $\chi^2(1) = 18.68$, $p < .001$, showing that women with high numeracy exhibited better gist comprehension of the trade-off compared to women with low numeracy. The odds of high-numerate women exhibiting better gist comprehension of the trade-off was 2.72 (95% CI, 1.73 to 4.29) times that of lower numerate women.

There was no interaction between format and numeracy on gist comprehension of the probability of side-effects, Wald $\chi^2(3) = 3.89$, $p = .273$. However, there was a significant main effect of numeracy, Wald $\chi^2(1) = 15.86$, $p < .001$. As with the gist comprehension of the trade-off, women with high numeracy were also more likely to exhibit higher gist comprehension of the probability of side-effects, compared to women with low numeracy. The odds of higher-numerate women exhibiting higher gist comprehension of the probability of side-effects was 2.68 (95% CI, 1.65 to 4.35) times that of low-numerate women.

For feeling informed, there was a main effect for numeracy, $F(1, 272) = 15.39$, $p < .001$, partial $\eta^2 = .05$. Women with high numeracy felt more informed (marginal means 4.19 ± .05) than women with low numeracy (marginal means 3.87 ± .06), a mean difference of .32 (95% CI, .16 to .48).

Numeracy – secondary outcomes

For experienced affect, there was a main effect for numeracy on Positive Affect, $F(1, 272) = 6.58$, $p = .011$, partial $\eta^2 = .02$. Women with low numeracy reported more Positive Affect (marginal means 2.54 ± .06) compared to women with high numeracy (marginal means 2.34 ± .05), a mean difference of .21 (95% CI, .05 to .37). For Negative Affect too, there was a significant main effect of numeracy, $F(1, 272) = 5.06$, $p = .025$, partial $\eta^2 = .02$. Women with low numeracy also reported more Negative Affect (marginal means 2.60 ± .11) than women with high numeracy (marginal means 2.29 ± .09), a mean difference of .32 (95% CI, .04 to .60). For the perceived affect scores on overwhelmed and worried, no significant interactions or main effects were demonstrated.

For risk perception regarding hormone therapy, there was a main effect for format, $F(4, 272) = 4.80$, $p = .001$, partial $\eta^2 = .07$. The pairwise comparisons showed a significant difference between format A (no probability information) and format C (visualised probability information without description) of .96 (95% CI, .27 to 1.66), p = .001. The risk perception for women presented with format A was higher (marginal means 7.38 ± .18) compared to the risk perception of women presented with format C (marginal means 6.41 ± .17). For risk perception regarding chemotherapy, there was a main effect for format, $F(4, 272) = 2.59$, $p = .04$, partial $\eta^2 = .04$. However, none of the pairwise comparisons was statistically significant.

Regarding preparation for decision-making, there was a main effect of numeracy, $F(1, 272) = 11.65$, $p = .001$, partial $\eta^2 = .04$. Women with high numeracy felt more prepared for decision-making (marginal means 3.90 ± .06) than women with low numeracy (marginal means 3.60 ± .07), a mean difference of .30 (95% CI, .13 to .48). Regarding uncertainty, evaluation of information, and decision certainty, there were no significant interactions or main effects of numeracy and format. Also, the effect of numeracy on the hypothetical treatment decision was not significant.

There was a main effect of numeracy on the realism scores, $F(1, 272) = 10.01$, $p = .002$, partial $\eta^2 = .04$. As in the first experiment, women with high numeracy (marginal means 7.80 ± .12) were

more likely to empathise with the hypothetical scenario than women with low numeracy (marginal means 7.21 ± .15), a mean difference of .59 (95% CI, .22 to .96).

Graph Literacy – primary outcomes

There was no significant interaction between format and GL on gist comprehension of the trade-off, Wald $\chi^2(4) = 3.80$, $p = .434$. However, we did find a significant main effect of GL, Wald $\chi^2(1) = 6.25$, $p = .012$. This effect showed that women with high GL exhibited better gist comprehension of the trade-off compared to women with low GL. The odds of high-GL women exhibiting higher gist comprehension of the trade-off was 1.78 (95% CI, 1.13 to 2.80) times that of low-GL women.

There was no interaction effect between format and numeracy on gist comprehension of the probability of side-effects, Wald $\chi^2(3) = 5.24$, $p = .155$. We did find a significant main effect of GL, Wald $\chi^2(1) = 25.12$, $p < .001$. The odds of high-GL women exhibiting better gist comprehension of the trade-off was 3.56 (95% CI, 2.17 to 5.86) times that of low-GL women, which means that women with high GL were more likely to exhibit better gist comprehension of the probability of side-effects compared to women with low GL.

For feeling informed, there was a main effect for GL, $F(1, 272) = 11.22$, $p = .001$, partial $\eta^2 = .040$. Women with high GL felt more informed (marginal means 4.23 ± .06) compared to women with low GL (marginal means 3.96 ± .05), a mean difference of .27 (95% CI, .11 to .43).

Graph Literacy – secondary outcomes

For experienced affect, there was a main effect for GL on Positive Affect, $F(1, 272) = 4.45$, $p = .04$, partial $\eta^2 = .02$. Women with low GL experienced more Positive Affect (marginal means 2.48 ± .05) than women with high GL (marginal means 2.31 ± .06), a mean difference of .17 (95% CI, .01 to .34). For overwhelmed, there was a significant effect of format, $F(4, 272) = 2.45$, $p = .047$, partial $\eta^2 = .04$. However, there were no significant differences in the

pairwise comparisons. For Negative Affect and being worried, no significant interactions or main effects were demonstrated.

For risk perception regarding hormone therapy, there was a main effect of format, $F(4, 272) = 3.53$, $p = .008$, partial $\eta^2 = .05$. The pairwise comparisons showed a significant difference between format A (no probability information) and format C (visualised probability information without description) of .79 (95% CI, .11 to 1.47), p = .002. The risk perception for women presented with format A was higher (marginal means 7.25 ± .17) compared to the risk perception of women presented with format C (marginal means 6.46 ± .17). For risk perception regarding chemotherapy, there was no significant interaction or main effect.

Regarding preparation for decision-making, there was a main effect of GL, $F(1, 272) = 6.61$, $p = .011$, partial $\eta^2 = .02$. Women with high GL felt more prepared for decision-making (marginal means 3.93 ± .07) than women with low GL (marginal means 3.71 ± .06), a mean difference of .23 (95% CI, .05 to .40). Regarding uncertainty and decision certainty, there were no significant interactions or main effects of GL and format. Also, the effect of GL on the hypothetical decision was not significant.

For evaluation of information there was a main effect of GL, $F(1, 272) = 4.12$, $p = .043$, partial $\eta^2 = .02$. Evaluation scores for women with high GL were more positive (marginal means 7.91 ± .15) compared to women with low GL (7.53 ± .12), a mean difference of .38 (95% CI, .01 to .76).

There was a main effect of GL on the realism scores, $F(1, 272) = 3.94$, $p = .048$, partial $\eta^2 = .01$. Women with high GL (marginal means 7.79 ± .15) were more likely to empathise with the hypothetical scenario than women with low GL (marginal means 7.42 ± .12), a mean difference of .37 (95% CI, .00 to .75).

Correlations between Health Literacy, numeracy and Graph Literacy

A chi-square test for association was conducted between HL and numeracy and between HL and Graph Literacy. There was no statistically significant association between HL and numeracy $\chi^2(1)$ =

1.39, p = .238; φ = -.07, p = .24 nor between HL and Graph Literacy $\chi^2(1) = 3.60$, p = .058; φ = -.11, p = .058.

References

1. Schwartz LM, Woloshin S, Black WC, Welch HG. The Role of Numeracy in Understanding the Benefit of Screening Mammography. Annals of Internal Medicine. 1997;127(11):966-72. doi: 10.7326/0003-4819-127-11-199712010-00003.

2. Okan Y, Janssen E, Galesic M, Waters EA. Using the Short Graph Literacy Scale to Predict Precursors of Health Behavior Change. Medical decision making : an international journal of the Society for Medical Decision Making. 2019;39(3):183-95. Epub 2019/03/08. doi: 10.1177/0272989X19829728. PubMed PMID: 30845893.
