# Supplementary material for: Presenting decision-relevant numerical information to Dutch women aged 50–70 with varying levels of health literacy: Case example of adjuvant systemic therapy for breast cancer
Source: PLoS One. 2024 Sep 3;19(9):e0309668. doi: 10.1371/journal.pone.0309668 (PMC11371237; doi:10.1371/journal.pone.0309668)
Supplement: S4 File — (PDF) [file pone.0309668.s004.pdf]

Supplemental Material 4 - Ordinal logistic regression for the interaction and main effects of format and health literacy on comprehension

	Outcome measure	Variables	Wald χ2	df	*p*
Experiment 1	Comprehension – gist	Interaction between health literacy and format	2.74	2	.254
		Health literacy	6.84	1	**.009**
		Format	.83	2	.660
	Comprehension - verbatim	Interaction between health literacy and format[a]	.108	2	.947
		Health literacy	1.32	1	.251
		Format	2.15	2	.342
Experiment 2	Gist comprehension of the trade-off	Interaction between health literacy and format	5.92	4	.206
		Health literacy	.61	1	.436
		Format	4.68	4	.322
	Gist comprehension side-effects probability	Interaction between health literacy and format	1.41	3	.703
		Health literacy	1.56	1	.211
		Format	1.17	3	.760

Note. If the interaction between health literacy and format was not significant, the model was run without interaction to examine the main effects of format and health literacy. [a]The model with interaction violated the assumption of proportional odds. Therefore, a multinomial logistic regression was conducted to test the interaction between format and health literacy on verbatim comprehension, which also showed no significant interaction, $\chi^2(8) = 12.35$, $p = .136$.
