# Supplementary material for: Presenting decision-relevant numerical information to Dutch women aged 50–70 with varying levels of health literacy: Case example of adjuvant systemic therapy for breast cancer
Source: PLoS One. 2024 Sep 3;19(9):e0309668. doi: 10.1371/journal.pone.0309668 (PMC11371237; doi:10.1371/journal.pone.0309668)
Supplement: Attachment — Submitted filename: Response to Reviewers 22-2-2024.pdf [file pone.0309668.s005.pdf]

Response to Reviewers

Reviewer #1: This paper reports a study which aimed to investigate the effect of survival rates and side-effects presentation formats on understanding and feeling informed and effects of health literacy using breast cancer therapy as an example. The study question is important and the study is interestingly designed. I do however have a few important comments for the authors to consider:

1. Overall, the writing could be clearer. For example, some of the sentences are quite long.

   RESPONSE: We thank the reviewer for this suggestion. We have made changes to the manuscript to increase the readability. In particular, we converted long sentences into shorter ones.

   - Line 55 ‘Several studies showed that visual formats improve both gist and verbatim understanding [6]. However, other studies found that visual formats are superior mainly for communicating gist messages and numerical formats for verbatim messages [4].’
   - Line 65 ‘However, only one study compared icon arrays and bar graphs, showing a 2-option icon array was best understood compared to bar graphs and 4-option visualisations [20]. In none of these studies a direct comparison with a non-visual format was made.’
   - Line 72 ‘However, the information on side-effects was limited and consisted of one side-effect only. This does not fully correspond to the variety of information on side-effects that is usually provided for cancer treatment such as adjuvant systematic treatment for breast cancer.’
   - Line 77 ‘To gain more insight into how to present decision-relevant information in the context of systemic adjuvant treatment for breast cancer, we co-created several presentation formats regarding benefit (i.e., survival rates) and potential harm (i.e., side-effects) with patients with breast cancer in a previous study [26]. To account for potential HL differences, this prior study also included patients with low HL.’
   - Line 97 ‘First, our information was similar to the complex information that can be provided in oncology practice, such as multiple side-effects with probability estimates with quite a large range. Previous studies simplified this information.’
   - Line 173 ‘The fact that we used likelihood estimates in a fairly wide range was driven by clinical reality. We wanted to investigate information formats that could be used in oncology practice and in the Netherlands (nor in most other countries, as far as we know) no exact point estimates were available.’
   - Line 360 ‘Contrary to what was described in our pre-registration, in both experiments the analyses with comprehension as outcome were performed with cumulative odds ordinal logistic regression with proportional odds (instead of ANOVAs). This was due to the ordinal nature of the comprehension variables with limited possible outcomes (i.e., ranging from 0-3 and 0-6).’
   - Line 527 ‘When examining comprehension, gist and verbatim comprehension were assessed using self-composed questions. This was due to an absence of validated questionnaires, as these concepts depend on the specific information presented.’

- Line 533 ‘One may argue that other, unassessed, gist representations can also be distilled from the information provided. For example, that the additional benefit of adjuvant systemic therapy is ‘relatively small’ or that even without additional treatment most women will still be alive in 10 years.’
- Line 539 ‘The questionnaire was translated to a reading level of up to sixth grade by a plain language expert and participants saw the information on screen while answering questions. Nevertheless answering our questions may have been difficult, especially for those with lower HL.’

2. Why did the authors choose breast cancer therapy as the case example? Could findings be different in another example? This is not entirely clear to me throughout.

   RESPONSE: This is an interesting question. This study was part of a larger consortium that explored the integration of personalized breast cancer estimates in Patient Decision Aids. Breast cancer is a disease for which multiple treatment options exist. There is relatively a large amount of data available on survival rates with different adjuvant systemic treatments, making it possible to build prognostic models. This makes breast cancer an interesting example to see how this data can be shared with patients to support shared decision-making. There are also developments in the field of personalized estimates in the context of other types of cancers, such as lung cancer, prostate cancer, and stomach and esophageal cancer. We think that findings can be relevant in those contexts as well. We also think that our results may be interesting for other cancers where adjuvant therapies are used. However, the results may not always be 100% generalizable to other contexts. For example, the prognosis of patients may differ across contexts as well as the treatment options available and the chances of survival related to those treatments. Also, the average age of the patient population can influence the way decision-relevant information should be presented. So although lessons can be learned from this study for other conditions, the specific context must always be considered.

   - We added to the Discussion (Line 543): ‘This study used adjuvant systemic therapy for breast cancer as a case example. However, more and more decision-relevant data are becoming available for other forms of cancer as well, such as lung cancer and stomach and oesophageal cancer. The results of the current study are important in presenting decision-relevant numerical information from these other cancers. However, the context and specific decision to be made should be taken into account. For example, the survival rates, available treatment options, prognosis, and the average age of the patient population can influence comprehension. Therefore, the results of this research can be used in developing presentation formats for other conditions, but user-testing within the specific context remains necessary’

3. There is a lot of information in the Methods – it is quite hard to follow. I appreciate all of the Tables and Figures, however is there any way this could be simplified? Consider collating all of it together as an Appendix (ie. the entire survey) and simplifying in the text to make it easier for those who are not familiar with these methods to follow.

   RESPONSE: We agree that the Methods section could benefit from changes to its structure to make it easier to follow. We also agree that there are a lot of Tables. For readability and understanding it does not seem desirable to move all the information to a supplemental. However, to ensure readability, we have moved the secondary

outcomes (Table 4) to the Supplemental and Figure 1 to the Results section. Further changes to the manuscript are listed below.

- We moved the description of HL measuring to the Participants subsection instead of the Design subsection. Line 221 'To ensure that approximately half of the participants had low HL, women's HL was assessed before the experiments with the Set of Brief Screening Questions (SBSQ) in Dutch, a self-reported measure with three questions measured on a 5-point scale (0=always, 4=never). The three questions were: (1) How often do you have someone help you read hospital materials?; (2) How confident are you filling out medical forms by yourself?; (3) How often do you have problems learning about your medical condition because of difficulty understanding written information? [31, 32]. Women who scored ≤2 on one of the questions were indicated as having lower HL. HL questions were posed together with the question if they had (or had had) breast cancer (exclusion criteria).'
- We have merged the subsections Design and Materials for a simpler structure. This also allowed us to reduce the overall amount of information. Line 108 'In two online randomised experiments, women viewed presentation formats with hypothetical information embedded in an online survey. Experiment 2 was performed after the first and contained new participants. Before data collection, we formulated hypotheses, shown in Table 1. This study was exempted from review by the medical research ethics committee of Amsterdam UMC, location VUmc (FWA00017598) following local regulatory guidelines/standards for human subjects protection in the Netherlands (Medical Research Involving Human Subjects Act). Participants provided written informed consent (online) after reading the study aim in the online survey. This study was pre-registered before data collection through the Open Science Framework on July 8th, 2021 https://osf.io/sxpjf/?view_only=cb702fb0aa904758bc2ce4a19abf0b74. Deviations from the pre-registration are indicated.

  The first experiment contained a 3 (survival rate format: text block – bar graph – icon array) x 2 (HL: low – high) between-subjects design. In the formats, three treatment options were presented: (1) no additional treatment; (2) hormone therapy; and (3) combination of hormone therapy/chemotherapy. The numerical information was an example of personalised information obtained from a prognostic model [33]. In co-creation with breast cancer patients, various survival rate visualisations were developed [26]. A bar graph and icon array seemed most appropriate in this context, although there were mixed results regarding the feelings evoked by icon arrays (i.e., some felt overwhelmed and others perceived them as more personal). To compare visualised data with textual numerical data, a text block format was included that did not visualize the numerical information but did use visual elements (e.g., three text blocks to indicate three options). See Fig 2 for the survival rate formats as used in experiment 1.

  We built on the best-understood survival rate format from experiment 1 in experiment 2. This survival rate format would be the same for all participants in experiment 2. This second experiment contained a 5 (side-effects format: no probability information – probability information in numbers without description – visualised probability information without description – probability information in numbers with accompanying description – visualised probability information with accompanying description) x 2 (HL: low – high) between-subjects design. The five side-effects presentation formats are described in Table 2 and S1. Fig 3 shows the format with the

most extensive information. The first format contained no probability information, which resembles how side-effect information is often presented to patients. The other formats contained probability information in numbers or a visualisation. Additionally, some formats contained contextual information (whether a side-effect disappears after treatment and whether something can be done about this side-effect). This need for contextual information was expressed by patients in the preceding co-creation sessions [26]. The fact that we used likelihood estimates in a fairly wide range was driven by clinical reality, as we wanted to investigate information formats that could be used in oncology practice and in the Netherlands (nor in most other countries, as far as we know) no exact point estimates were available. So in the end, we developed a visualisation inspired by the results of the co-creation sessions (i.e., a horizontal bar graph), but also based on the available information (i.e., whether side-effects occur in 1-10 or more than 10 out of 100 women). The newly developed visualisation was pre-tested with eight women. An oncologist (I.R.H.M.K) reviewed the medical content of both experiments to ensure accuracy and compliance with practice in encounters with patients.'

4. Following on from that, there are a lot of tables – I think some need to be reduced (suggest in the Methods as opposed to the Results).

   RESPONSE: While we agree that there is a lot of information in the Tables, we think the tables are necessary to fully understand both experiments. However, we have moved Table 4, which describes the secondary outcome measures, to the supplemental.

5. In the first Figure make clear Experiment 1 and 2.

   RESPONSE: Unfortunately, we do not fully understand this comment. What would the reviewer like to see? The Figure already states experiment 1 and experiment 2. How would the reviewer like to see this differently? Note: we have moved this Figure to the Results section so it is now Figure 3.

6. The figures are coming up blurry – please check quality.

   RESPONSE: We have now uploaded our figure files to the Preflight Analysis and Conversion Engine (PACE) digital diagnostic tool as recommended by PLOS. The Figure files should now meet the PLOS criteria and should no longer be blurry.

7. While the Discussion is well written, the conclusions could be much stronger. What does this mean for current policy and practice – what else could be done to help with this or future research in regard to health literacy?

   RESPONSE: We thank the reviewer for this suggestion and have elaborated our Conclusion.

   - Line 585: 'No evidence was found for a medium effect size in comprehension when presenting decision-relevant numerical information to patients in either a well-designed text block, bar graph, or icon array that all adhered to risk communication best practices. Providing patients with visualisations might not necessarily yield an

advantage over providing structured numerical information. These results have practical implications, for example, for patient decision aid developers. The fact that visualizing numerical information is not a magic bullet is relevant when developing patient decision aids. Furthermore, the results of this study show that a deeper understanding of how to present numerical and context-specific information about side-effects seems needed. Especially for patients with lower information processing skills this is important. They understood the information less well and experienced more negative affect when receiving side-effect information.'

Reviewer #2: This study endeavors to create and evaluate visual aids conveying both survival rates and the probabilities of side effects associated with a specific breast cancer treatment. The design of visual aids adhered to state-of-the-art recommendations and involved consultations with the oncologist and patients. The manuscript maintains a robust theoretical foundation, exhibiting transparency through preregistration, comprehensive outcome reporting, and inclusion of statistical analyses, even when non-significant.
However, to enhance the manuscript's accessibility for other researchers, I propose several improvements.

Firstly, supplementing details in the supplementary materials compensating for the lack of a dataset would increase the potential utility of the research in future meta-analyses (e.g., by incorporating a matrix of correlations and reliability analyses for all measures in the supplementary materials).

RESPONSE: We thank the reviewer for pointing out the importance of data availability for future use in meta-analyses. We cannot make the data publicly available because we did not specifically request this in the Informed Consent. And our local regulations require this when making the data publicly available. However, we can make our data available to interested researchers conducting a meta-analysis. To ensure that the data can also be retrieved in the future, we have added the details of the department secretariat in addition to the correspondence details of the first author. They can approach the head of the department and according to local rules, the department head is responsible for the research data. In this way, we hope to be able to guarantee long-term stability and availability of data.
We have added Cronbach's Alpha of the primary outcome measure Feeling informed to the footnote of Table 3. And we have added the Cronbach's Alphas of the secondary outcome measures in S2.

- The Data Availability Statement: Data cannot be shared publicly because the Ethics Committee approved the collection and analysis of the data for the specific study only. The Ethics Committee requires that the data collected remains securely stored and not be shared publicly. Researchers who meet the criteria for access to confidential data can contact i.vanstrien@amsterdamumc.nl to request the data. In addition, the data can also be requested from the head of the Amsterdam UMCs Public and Occupational Health department via the departments secretariat Div10-POHsecretariaat@amsterdamumc.nl
- Line 306: 'Note. The Cronbach's Alpha of the Feeling informed scale is .843.'
- Please, see S2 for the Cronbach's Alphas of the secondary outcome measures.

Additionally, a more comprehensive dropout analysis for each experimental condition, along with updates to Figure 1, is recommended, considering potential variations in emotional responses across conditions. Figure 1 would also benefit from additional information on the assignment method for experiments.

RESPONSE: We agree with the reviewer that it could be informative to have a more extensive dropout analysis. Unfortunately, we do not have information about the dropouts within the various conditions. This is because a panel was used and we agreed that we would only receive the completed questionnaires. However, as no differences were found in emotional responses between the different information formats, we believe that this did not play a significant role. Regarding the assignment method, randomization with quotas on health literacy was used to assign women to the first or second experiment. The women were then assigned to a presentation format through a technical process in the survey. We have moved the information regarding the randomization in the manuscript and added it to the Figure.

- Line 229 '<u>Randomisation with quotas on HL was used to assign women to the first or second experiment and to assign them to a presentation format.</u>'
- Note added to the Figure (Line 377) '<u>Randomisation with quotas on HL was used to assign women to the first or second experiment and to assign them to a presentation format.'</u>

Furthermore, I suggest conducting analyses controlling for numeracy, graph literacy, and education to ensure that health literacy indeed predicts the outcomes.

RESPONSE: We thank the reviewer for this suggestion. However, the focus of our manuscript is to investigate the effect of survival rates and side-effects presentation formats on comprehension and 'feeling informed'. We thereby wanted to investigate whether there are differential effects among women with higher and lower Health Literacy. So we focused on the comprehension of the information and included Health Literacy as a possible moderator in the effect. In addition, with the current data we cannot perform ordinal logistic regression with all the listed variables. The number of participants is too low for such an analyses, which would result in too many cells with zero or limited frequencies.

As the study could evoke negative emotions, please provide more details about measures that the authors used to ensure the well-being of the subjects.

RESPONSE: We agree with the reviewer that the information may provoke negative reactions. For this reason, we have stated at various moments in the questionnaire that the medical information is an example and does not relate to the participant herself. We have now added this to the manuscript.

- Line 249 <u>'To emphasize that the medical information did not relate to the participants themselves, it was stated several times in the survey: '*Please note: the information is NOT real information. The information is an example. It is not about your own medical situation.''*</u>

I recommend, adding more information regarding the numeracy measure, including the reference, psychometric properties, and relationships with other measures (the measure is introduced only briefly in the supplementary materials).

RESPONSE: Although the focus of the manuscript was on Health Literacy, the information on the numeracy measure could indeed be more comprehensive. We added information about the questions regarding the numeracy measure in S3. However, the survey was conducted in Dutch and to our knowledge no psychometric properties of the translated Dutch questions are known. To provide more insight into the number of correct answers to the three numeracy questions, we have included this information per experiment in S3. Also, the association between Health Literacy and numeracy is reported in S3.

- S3 '<u>Numeracy was assessed using the three mathematical questions of Schwartz et al. [1] about probabilities and ratio concepts. The questions are 'Imagine that we flip a fair coin 1,000 times. What is your best guess about how many times the coin would come up heads in 1,000 flips?', 'In the Big Bucks Lottery, the chance of winning a $10 prize is 1%. What is your best guess about how many people would win a $10 prize if 1000 people each buy a single ticket to Big Bucks?', and 'In ACME Publishing Sweepsakes the chance of winning a car is 1 in 1,000. What percent of tickets to ACME Publishing Sweepsakes win a car?'.</u>'
- S3 '<u>In the first experiment, the first numeracy question was answered correctly by 66.7% of the total of 219 participants. The second question by 73.1% and the third question by 38.4%.</u>'
- S3 '<u>In the second experiment, the first numeracy question was answered correctly by 63.1% of the total of 282 participants. The second question by 70.2% and the third question by 39.7%.</u>'
- S3 Regarding the first experiment: 'A chi-square test for association was conducted between HL and numeracy and between HL and Graph Literacy. There was a statistically significant association between HL and numeracy $\chi 2(1) = 3.854$, $p = .050$; $\varphi = -1.333$, $p = .050$. There was no statistically significant association between HL and Graph Literacy $\chi 2(1) = 1.379$, $p = .240$; $\varphi = -.079$, $p = .240$.'
- S3 Regarding the second experiment: 'A chi-square test for association was conducted between HL and numeracy and between HL and Graph Literacy. There was no statistically significant association between HL and numeracy $\chi 2(1) = 1.390$, $p = .238$; $\varphi = -.070$, $p = .238$ nor between HL and Graph Literacy $\chi 2(1) = 3.600$, $p = .058$; $\varphi = -.113$, $p = .058$.'

1. Schwartz LM, Woloshin S, Black WC, Welch HG. The Role of Numeracy in Understanding the Benefit of Screening Mammography. Annals of Internal Medicine. 1997;127(11):966-72. doi: 10.7326/0003-4819-127-11-199712010-00003.

Finally, A revised title reflecting the exclusive focus on female subjects aged 50-70 and the absence of real breast cancer patients would enhance accuracy.

RESPONSE: We revised the title into 'Presenting decision-relevant numerical information to <u>Dutch women aged 50-70</u> with varying levels of Health Literacy: case example of adjuvant systemic therapy for breast cancer'.

Reviewer #3: This manuscript reports an online experimental study conducted among women in the Netherlands. The study investigates important questions that are broadly relevant for determining the best formats to use in health information provision. The manuscript is generally clear and well written. Below, I have indicated a few points where I think information is missing and some suggestions to consider to improve the manuscript's presentation and English expression.

DATA AVAILABILITY

1. Information from the manuscript submission system says the "study was exempted from review by the medical research ethics committee". However, it also says: "Data cannot be shared publicly because the Ethics Committee approved the collection and analysis of the data for the specific study only. The Ethics Committee requires that the data collected remains securely stored and not be shared publicly." To me, these statements seem contradictory. The authors need to clarify.

> RESPONSE: We understand that this may cause confusion. Ethical approval was obtained from the Medical Ethics Review Committee. This committee also decides whether the Dutch Medical Research Involving Human Subjects Act (WMO) does apply to a study. If the WMO applies, there will be an additional procedure in which the study must comply with other specific guidelines. The Medical Ethical Review Committee assessed our study and determined that the Dutch WMO did not apply to our research. We clarified this in the Manuscript.
>
> - Line 112 'Ethical approval was obtained from the medical research ethics committee of Amsterdam UMC, location VUmc (FWA00017598). <u>The Dutch Medical Research Involving Human Subjects Act (WMO) did not apply</u>.'

INTRODUCTION

2. Page 5 line 97-98. This sentence is too long and awkward. I suggest breaking it down into 2 sentences to convey the ideas that (a) unlike previous studies, we designed info to reflect practice; (b) specifically, in practice info is complex and includes… etc.

> RESPONSE: We thank the reviewer for this suggestion and we have split it into two sentences.
>
> - Line 97 'First, our information was similar to the <u>complex</u> information that can be provided in oncology practice, such as multiple side-effects with probability estimates with quite a large range. <u>Previous studies simplified</u> this information.'

METHODS

3. Page 8 line 145. I would save the presentation and mention of Figure 1 until the Results section.

RESPONSE: We have moved Figure 1 to the Results section. This Figure has now become Figure 3.

4. Page 8 line 149-156. I think the information about the sample size calculation belongs in the Data Analysis section.

RESPONSE: We moved the information about the sample size calculation.

- Line 356 'An a priori power analysis was performed based on a 3x2 (experiment 1) and a 5x2 factorial ANOVA design (experiment 2) with interaction-effect. With a medium effect size (ES) of .25 (Cohen's f) [33], alpha of .05, and power of .90, the required sample sizes were 210 and 260. A medium effect size was chosen from a pragmatic point of view as we wanted to formulate recommendations for practical implementations (e.g., PtDAs).'

5. Please specify when and how participant demographics and background characteristics were collected (i.e., age, education level, medical background, medical knowledge).

RESPONSE: We agree that this information could be included in the manuscript. We have added it to the Methods section.

- Line 293 'Regarding participants' demographic characteristics, the age and education level were already known to the panel. The survey included questions about the medical knowledge and medical education of participants.'

6. Page 9 line 163. "empathise with this situation" may be the accurate direct translation from Dutch, but reading the scenario presented in Figure 2, I suggest it would be more appropriate to say "imagine themselves in the hypothetical situation" (as per Page 15 line 259).

RESPONSE: We have adopted this suggestion.

- Line 247 'Participants received hypothetical but realistic information about a woman with primary hormone-sensitive/Her2Neu-negative breast cancer and were asked to imagine themselves in the hypothetical situation.'

7. Table 4. The abbreviations PA and NA are defined in the text, but they should also be in a footnote to the table. Also, this label is missing from Overwhelmed and Worried. After reading further, I now realise this is because these 2 terms were added to the original PANAS items. However, I think it would be helpful to use an asterisk or something and put a brief explanation in a footnote to the table.

RESPONSE: We have added footnotes to the Table. It is important to note that this Table has been moved to a supplemental and can be found in S2.

- S2 'Notes. [a]NA = Negative Affect. [b]PA = Positive Affect. [c]Worried and Overwhelmed were added to the original PANAS items.'

8. Secondary outcome measures. To avoid confusion with “decision uncertainty”, I suggest renaming the single item “decision confidence” instead of “decision certainty”.

RESPONSE: We have included this in the manuscript.

- Line 93 ‘Since presentation formats of decision-relevant information can also influence patients’ intentions and evaluations [17], we explored effects on several secondary outcomes: affect, decision, <u>decision confidence</u>, and evaluation of information.’
- Tables 5, 6, and S2: ‘Decision <u>confidence</u>’.
- Line 335 ‘Decision <u>confidence</u>: One item about how confident one was about the decision (1=not confident at all, 10=very confident).’
- Line 466 ‘For the secondary outcomes decision uncertainty, preparedness for decision-making, evaluation of information, and decision <u>confidence</u>, there were no interactions or main effects.’

RESULTS

9. Table 5. A cell in the middle of this table – “20 (37.0)” – is missing the “%” sign.

RESPONSE: We have added the % sign.

10. Did the authors check for any significant differences between groups on any of these characteristics? If there were differences, should these have been controlled for in analyses?

RESPONSE: For both experiments we checked whether there were significant differences between the participants assigned to the various formats on the characteristics listed in Table 4 (age, education, HL, medical education, and medical knowledge). No differences were found. We had not mentioned this in the manuscript, so we have added it now.

- We added to the footnote of Table 4 <u>‘Note. We checked whether there were differences in the listed characteristics between the participants of the different formats. There were no significant differences in either experiment.’</u>

11. Page 18 line 297. “HL was neither associated” should be “Nor was HL associated”.

RESPONSE: We changed the wording in the manuscript.

12. Page 19 line 316. Similarly, “HL neither influences” should be “Nor did HL influence”.

RESPONSE: We changed the wording in the manuscript.

13. Figure 4. I think the authors should consider whether this information would be better conveyed via a bar chart, because there are distinct groups/conditions being compared (as opposed to different points in time).

RESPONSE: We thank the reviewer for this suggestion and have now shown Figure 4 in a bar chart.

DISCUSSION

14. Page 26 line 449. “this limitation does not seem to have occurred” doesn’t seem quite right; perhaps replace with something like “this limitation was not consequential” or “this limitation ultimately did not influence our findings”.

RESPONSE: We changed the wording in the manuscript.

- Line 566 ‘However, since no major effects were found, this limitation ultimately did not influence our findings.’

15. Page 26 line 450. The acronym IPDAS should be explained.

RESPONSE: We agree that the acronym needs to be explained. We have added it to the manuscript.

- Line 568 ‘The effect sizes of the studies on which the International Patient Decision Aid Standards (IPDAS) collaboration’s recommendation to use visualisations is based are generally small to moderate’.

16. Page 26 line 456. “while ordinal logistic regression analyses were performed” – I suggest instead “whereas ultimately ordinal logistic regression analyses were performed”.

RESPONSE: We have corrected this in the manuscript.

- Line 574 ‘Moreover, initial power calculations were based on factorial ANOVA designs, whereas ultimately ordinal logistic regression analyses were performed.’
