# Supplementary material for: Presenting decision-relevant numerical information to Dutch women aged 50–70 with varying levels of health literacy: Case example of adjuvant systemic therapy for breast cancer
Source: PLoS One. 2024 Sep 3;19(9):e0309668. doi: 10.1371/journal.pone.0309668 (PMC11371237; doi:10.1371/journal.pone.0309668)
Supplement: Attachment — Submitted filename: Response to Reviewers May 2024.docx [file pone.0309668.s006.docx]

Dear Editor,

Thank you for the opportunity to resubmit our manuscript entitled *‘Presenting decision-relevant numerical information to Dutch women aged 50-70 with varying levels of health literacy: case example of adjuvant systemic therapy for breast cancer*’ (PONE-D-23-32983R1). We have addressed all the outstanding points as described below. Each comment is described and followed by our response. In our answers, changes to the manuscript are underlined. The main changes in the revised manuscript itself are indicated by tracked changes. The line numbers in our responses refer to the line numbers in the tracked changes manuscript.

Additional Editor Comments:
Dear Authors,
Thank you for submitting a revision that has addressed the reviewers’ concerns well!
My outstanding points are listed below:

1) The presentation formats fit for groups of differential education, numeracy, or health literacy is an important target. However, an argumentation is required. Why do you expect – according to which theory of health literacy – that people with different levels of health literacy would respond differently to a given piece of health information? For instance, HL-EU substantially refers to the perceived ability to seek, to find, and apply health information (besides evaluating and comprehending). For instance, the sentence in l.39-40, according to which skills related to information processing could be captured by HL is too speculative. The introduction needs to derive the health-literacy-expectations from the literature. Why should which concept of HL produce which type of differences given formats? This also may help explain why HL made no difference in comprehension in Experiment 2.

RESPONSE: We thank the editor for this excellent comment. We agree that more elaboration of the HL concept used in our study is useful. In our line of thinking/expectations, those who have more difficulty with evaluating and comprehending complex medical information -including the numerical aspects- were thought to benefit more from visual presentation formats designed to reduce cognitive effort/to increase intuitive/affective meaning of that information. As such, we wanted to focus on people who differ in those (perceived) evaluation/comprehension skills and not so much on (perceived) abilities in information seeking and finding. We agree that the sentence in line 39-40 was too speculative. We have clarified this in the manuscript.

- Lines 39-51. ‘However, understanding medical probability information is greatly influenced by patient skills related to information processing, such as the ability to derive meaning from abstract information and factual evaluation and appraisal of information [5]. Such information processing skills are captured in concepts such as health literacy (HL), which is mainly about accessing, understanding, and using health information [6]; numeracy, which is about understanding and using numbers and probabilities; and graph literacy (GL), which concerns the ability to understand graphically presented information [7]. Concerning HL there is a variety of conceptualizations. While some conceptualizations focus on a broad and holistic view of HL that also includes, for example, searching for information or assessing the reliability of information [e.g., 8], other conceptualizations focus more on specific aspects of HL, such as the literacy aspect [e.g., 9]. In this study, the focus is on understanding and interpreting abstract health information. Therefore HL is conceptualized as an individual ability in which understanding and interpreting health information is central.

  2) Your descriptive statistics even with a higher powered sample let nobody expect to find medium effects on comprehension depending on health literacy. Provided an argumentation for an interaction with health literacy, this leads to discuss
  a. the stimuli: The icon array and the bar chart in Experiment 1 have a small but relevant error how visualisations are labelled: The legend labels Hormone therapy and Chemotherapy in option 2 and 3 although correctly would be Surgery and Hormone Therapy and Surgery and Chemotherapy, respectively. A scientist may perceive this difference trivial, but actually this misspecification can create confusion in laypeople who aim to understand the legend, particularly if they are not really aware about that they might have already undergone surgery. Items referring to Hormone and Chemotherapy (verbatim!) could bring the visualisation formats at disadvantage. On the other hand, it would be sufficient to read (in Experiment 1) the statement “Alive after 10 years: XX%” – this allows for correct responding to any item and this is constant across presentation conditions. So, why to expect any difference? One could say, the graphs did not mislead them.

RESPONSE: We thank the editor for this attentiveness. We have tried to develop the most optimal visualisations based on co-creation, user testing, and existing guidelines on probability communication. For example, the co-creation sessions revealed that it is important for women that it is immediately clear what the effect of an additional treatment is. Therefore, the legend only mentions this additional effect, something the women in the co-creation sessions and user tests seemed to understand. An aspect also mentioned in the guidelines for communicating probability information is that it must be ensured that people have to make as few mental calculations as possible. This has resulted in the sentence 'alive after 10 years: XX' being included above the visualisations. However, because the survey questions focused on understanding the core information, the questions may not have been best suited to detect differences between the formats. We have now added this to the discussion.

- Lines 440 – 443 ‘It is also possible that despite developing the visualisations in co-creation with the target group, the visualisations were still not optimal. For example, women who were particularly focused on the visualisation and the legend may not have noticed that all the options were about someone who had already undergone surgery.’
- Lines 496 – 500 ‘It should be noted that the comprehension questions were mainly aimed at understanding the core message of the information. This may have resulted in the comprehension questions in Experiment 1 in particular being too easy and not necessarily the most focused on discovering differences between the formats. Therefore, the comprehension questions themselves may also have contributed to the lack of effects.’

b. the participants: Have they just not taken deep notice of the material and answered the questions correctly anyway? [e.g., the gist comprehension questions] Where attention checks for compliant responding included [only three for “poor data quality”, e.g. straightliners, excluded?]? I comprehend the power calculation, but the cells with low literates in the end are not all sufficiently powered, even assuming highly compliant participants.

RESPONSE: We agree that in online experiments such as ours, whether or not respondents complete the questions attentively can play a role. As mentioned in lines 280-281 the panel did examine the data for potential inattentive responders and poor data quality. The panel performed quality checks on open answers, consistency of answers, straight-lining (i.e., the same answer option is chosen throughout a series of statements), and completion time. However, it is possible that participants paid less attention to the information due to the hypothetical scenario. We have therefore added this to the limitations section.

Regarding the group size of the lower health literate, this is indeed a limitation of the study. We also acknowledge this is our strengths and limitations section in lines 538-543.

- Lines 523-524 ‘It may be that respondents paid less attention to the information due to the hypothetical scenario.’

c. the measurement. You did pretesting with 30 low-literate participants, but what has been learned? Items with more than 90% correctness in their group did not enter the main survey, anything else on discriminability? Probably the three gist comprehension tasks were too easy for guessing people – many people would expect more treatment more benefit. What are the internal consistencies of comprehension gist and comprehension verbatim and comprehension combined?

RESPONSE: We indeed pretested the questions of the first experiment among 67 women of whom 30 were low health literate. We looked at the number of correct answers and in addition, after each set of questions, respondents had the opportunity to comment on the previous questions. For example, they could indicate that a question was not clear or that there were too many questions. For example, there were six gist questions in the pre-test, but some respondents wondered whether they were real questions or whether they were trick questions because there were so many items that tried to capture more or less the same. That was an extra reason to remove the three gist questions that scored high, i.e., ≥90% correct. Regarding the verbatim questions, there were initially also six questions, but respondents commented that it felt like a math exam. That was also an extra reason to remove the two questions that scored high, i.e., ≥90% correct. Respondents also thought that the questionnaire as a whole was too long and that is why we omitted some questions. For example, we reduced the number of questions for the perception of treatment effect from six to two and only included the questions that were about the comparison of hormone treatment versus no additional treatment and the comparison of hormone treatment versus hormone treatment and chemotherapy. Furthermore, minor textual adjustments were made based on the open comments. We have now explained this more clearly in the manuscript and we have added to the discussion that the comprehension questions, particularly in the first experiment, may have been too easy and may not have been sufficiently focused on finding differences between the formats.

The internal consistencies of the comprehension scales are added to Table 3. No combined score for the gist and verbatim comprehension has been calculated. As outlined in the introduction, gist and verbatim comprehension are two different concepts that can have different effects on the understanding of visualisations.

- Lines 236-241 ‘To avoid ceiling effects in the experiment and to take into account respondents' comments that the number of questions made it feel like a math exam, we selected comprehension questions that were answered correctly by ≤90% of women with lower HL. In addition, the number of questions was reduced because respondents indicated that the questionnaire was too long and minor textual adjustments were made if respondents indicated that the question or response category was not clear.’
- Lines 496-500 ‘It should be noted that the comprehension questions were mainly aimed at understanding the core message of the information. This may have resulted in the comprehension questions in Experiment 1 in particular being too easy and not necessarily the most focused on discovering differences between the formats. Therefore, the comprehension questions themselves may also have contributed to the lack of effects.’
- Lines 246-248 ‘The Kuder-Richardson Reliability Coefficient for Experiment 1 is .71 for gist comprehension and .57 for verbatim comprehension. For Experiment 2 the Kuder-Richardson Reliability Coefficient is .44 for gist comprehension trade-off and .67 for gist comprehension side-effects probability.’

d. the analysis. Have you considered format analysis across all items (simple comprehension sum score)? Have you considered a sensitivity analysis excluding those, who respond “I don’t know”?

RESPONSE: As also indicated in the answer above, we assume that gist comprehension and verbatim comprehension measure different aspects of comprehension. That is why we did not want to calculate a total score.

We added the answer category 'I don't know' to prevent people who did not know the answer from filling in a random answer. It was mandatory to fill in an answer, so that could lead to respondents guessing the answer. Because the comprehension questions are about measuring understanding, we believe that respondents who answered 'I don't know' should not be excluded.

3) Please refer not only to shared decision making but to the goal of health communication enabling informed decisions according to evidence-based medicine, Western health system standard. Particularly, informing patients about benefits and harms is one of many rules according to established guidelines on how to design health communication (e.g., .

RESPONSE: We agree with the editor that this elaboration is useful. Accordingly, we have made adjustments in both the abstract, introduction, and discussion.

- Lines 3-4 ‘If communicated adequately, numerical decision-relevant information can support informed and shared decision-making.’
- Lines 32-34 ‘Informing patients about benefits and harms of different options is one of the key principles in health communication regarding informed and shared decision making (SDM) [1, 2].’
- Lines 424-426 ‘In this study, we investigated the effects of several (visual) presentation formats to present decision-relevant numerical information (i.e., survival rates and side-effects) to patients in support of informed and shared decision making (SDM).’

4) Also, how did you arrive at subscale of decisional conflict (instead of the full assessment?) Could you derive in the introduction why it is relevant to assess how someone could have felt informed?

RESPONSE: The Decisional Conflict scale consists of five subscales. In the second experiment, we used the Informed subscale as a primary outcome measure and the Uncertainty subscale as a secondary outcome measure. We have not included the other subscales because they concern value clarity, support from others, and the effectiveness of the decision. Because our experiment did not include explicit value clarification exercises nor was it presented in a context in which support from others could have played a role, these subscales did not seem relevant to our study. Instead, we used the six items of the preparedness for decision-making scale which assess the extent to which the information has been supportive. The rationale was that the presentation of decision-relevant information could help people feel more prepared for decision-making.

The secondary outcome measures such as feeling informed and the evaluation of the information were included because we know that decision-relevant information can have a different influence on such measures compared to the influence on comprehension (Trevena et al., 2021). We have added the following to the introduction:

- Lines 119-123 ‘In addition, we also assessed the perception of the treatment effect in the first experiment and risk perception regarding additional treatment in the second experiment. Because the information presented was intended to support decision-making, we also included decision uncertainty and the extent to which the information contributes to the perceived preparedness for decision-making in the second experiment.’

5) Abstract
a. “When communicated adequately..”
b. Capitalisation of shared decision making and health literacy seems unusual
c. When high/low …. Perhaps better expressed “depending on their”….
d. Probability information in numbers/visualisations … Perhaps better expressed “numbers with or without…” Numbers accompanied also visualisations here.

RESPONSE: We thank the editor for his careful reading. We have made changes to the abstract and where appropriate to the rest of the manuscript, e.g., omitting the capitalization.

- Lines 3-5. ‘If communicated adequately, numerical decision-relevant information can support informed and shared decision making. Visual formats are recommended, but which format supports patients depending on their health literacy (HL) levels for specific decisions is unclear.’
- Lines 98-100 ‘In addition, previous research has not investigated which format best suits people depending on their information processing skills.’
- Lines 13-17. ‘Experiment 2 had a 5 (side-effects format: no probability information – probability information in numbers with or without a visualisation – probability information in numbers with or without a visualisation accompanied by a description of the side-effects) x 2 (HL: low – high) design.’

6) Introduction; generally: the impression of specific visualisation that outperforms no visualisation should be avoided, because state of evidence is that different presentation formats are beneficial for different problems and different dialog groups. Please leave it in a format comparison, as you analysed it, bar vs. text and icon vs. text.

RESPONSE: We agree with the editor and did not want to create this impression. We have made an adjustment to the introduction and hope that this has removed the wrong impression.

- Lines 53-57 ‘Visual formats are recommended for certain information types, such as line graphs to show trends over time and bar graphs to compare multiple outcomes [11], although studies have not always compared such formats to a format without visualisation. Whether a visualisation has added value and which visualisation is best to use depends on the purpose of the communication [12] and the target group [13].‘

a. L.49… reduced by conveying the part-to-whole relationship [11].

RESPONSE: We have made this adjustment

- Line 61-63 ‘Furthermore, patterns are more visible, comparing multiple options is easier [16, 17], and denominator neglect (i.e., tendency to pay more attention to numerators than denominators) can be reduced by conveying the part-to-whole relationship [18].’

b. L.62… please be more explicit what is meant by general recommendations. There is evidence for different formats and decision problems, but do you mean guidelines?

RESPONSE: What we meant here is that there are recommendations for probability communication, but there is no clarity about which format should be used in which situation. We have tried to clarify this in the manuscript.

- Lines 75-78. ‘Although visual formats are generally mentioned in recommendations for probability communication, e.g., International Patient Decision Aid Standards (IPDAS) [4], due to variations in study design and outcome measures, there is no consensus on which format should be used in which situation [4, 24].’

c. L.61-l.76: some studies could be considered that compared communication formats for medical evidence (with and without text control) with regard to knowledge/comprehension, also with regard to education and health literacy – references below; you may find further literature, if you review literature on the health communication of benefits and harms

RESPONSE: We thank the editors for these suggestions. We have further elaborated on this in the manuscript based on the suggested literature.

- Lines 75-87 ‘Although visual formats are generally mentioned in recommendations for probability communication (e.g., IPDAS) [4], due to variations in study design and outcome measures, there is no consensus on which format should be used in which situation [4, 24]. For example, a study about the presentation of benefits and harms of cancer screening showed no differences in understanding fact boxes with numbers and fact boxes with icon arrays [25]. However, the decision to undergo cancer screening versus cancer treatment is arguably different. In the context of adjuvant systemic breast cancer treatment, previous studies of visual formats of survival rates (i.e., icon arrays and bar graphs) have been conducted [26-28]. However, only one study compared icon arrays and bar graphs, showing a 2-option icon array was best understood compared to bar graphs and 4-option visualisations [27]. In none of the studies in the context of adjuvant systemic breast cancer treatment, a direct comparison with a non-visual format was made.’
- Lines 88-97 ‘When studies presented information about side-effects of cancer treatments or treatments to reduce the cancer risk, the probability of side-effects was typically not presented visually [e.g., 29, 30-32]. One study examined two visual side-effect formats, i.e., a bar graph and an icon array, and found no differences in understanding. However, no information about the benefits was presented and no comparison with a textual condition was made [33]. One study used a bar graph to display probabilities of side-effects in addition to text in the context of medication to reduce the risk of developing cancer; participants were found to be more accurate when viewing this bar graph compared to text alone [15]. However, the information on side-effects was limited and consisted of one side-effect only.’

d. What is meant by decision (l.95) for women without BC from the general population (hypothetical decision, intention?).

RESPONSE: The editor is right, we do indeed mean the hypothetical decision and have adjusted this in the manuscript.

- Lines 116-119 ‘Since presentation formats of decision-relevant information can also influence patients’ intentions and evaluations [12], we explored effects on several secondary outcomes: affect, hypothetical decision, decision confidence, and evaluation of information.’
- Line 277: ‘*Hypothetical decision*: One question about participants’ choice after seeing the information.’
- Lines 306-307 ‘Likewise, due to the categorical nature of the outcome ‘hypothetical decision’, the effects of format and HL on this outcome were analysed using chi-square tests of association.’
- Tables 5 and 6 ‘Hypothetical decision’
- Lines 363-364 ‘Also, neither format, χ^2^(4) = 6.152, *p* = .188, nor HL, χ^2^(2) = 3.798, *p* = .150, were associated with the hypothetical decision.’
- Lines 419-421 ‘The effects of format and HL on the hypothetical decision were also not significant, nor were interactions or main effects regarding the realism check.’
- Supplemental 2 and 3 ‘Hypothetical decision’

e. Experiment 1 and 2 should be capitalised throughout the manuscript

RESPONSE: We have adjusted this throughout the manuscript and in Supplementals 2 and 3.

7) Method
a. Please mention that it is a convenience sample, not representative for Dutch women from 50-70.

RESPONSE: We have added this to the manuscript.

- Lines 188-189 ‘In both experiments, we used a convenience sample of women from the general Dutch population aged 50-70 years without a history of breast cancer.’

b. Move the SBSQ to the Measures section

RESPONSE: We report the SBSQ in the Participants section because the SBSQ was measured prior to the experiments and used to assign respondents to experiments 1 or 2. However, we have now also added this information in the Measures section and we have moved the three questions themselves from the Participants to the Measures section.

- Lines 221-226 ‘Participants' HL was measured before both experiments with the SBSQ in Dutch, a self-reported measure with three questions measured on a 5-point scale (0=always, 4=never). The three questions were: (1) How often do you have someone help you read hospital materials?; (2) How confident are you filling out medical forms by yourself?; (3) How often do you have problems learning about your medical condition because of difficulty understanding written information? [40,41].’

c. Why SBSQ, please explain, given so many others?

RESPONSE: We agree with the reviewer that there are many instruments for measuring health literacy, which also has to do with the different ways in which HL is conceptualized. As we have now explained in our manuscript, in response to the editor's first comment, we conceptualized HL as an individual ability in which understanding and interpreting health information is central. With this conceptualization, a questionnaire such as the HLS-EU, which also includes searching and assessing the reliability of information, is less suitable. In contrast, the SBSQ is consistent with this view of HL because it focuses on understanding health information. However, a questionnaire such as the NVS is also in line with our view of HL. We had therefore both administered the NVS-D and the SBSQ during the pretest. However, because people reacted negatively to the NVS-D and thought it was a math exam and because we also assessed numeracy as a separate concept in the experiments (see the information in Supplemental 3), we ultimately did not use the NVS-D. We have now elaborated on this in the manuscript.

- Lines 226-229 ‘The SBSQ was chosen as a measure for HL because it fits our view of HL in this study, namely as an individual ability in which understanding and interpreting health information is central. Moreover, in our pre-test, people responded negatively to the NVS-D and thought it resembled a math test.’

d. Though mentioned in the text, I cannot recognise quotas from Fig. 3.

RESPONSE: Indeed, the quotas are not shown in Figure 3. The quotas were used by the panel to ensure that there were enough participants with low HL in both experiments. Approximately the same number of participants with low and high HL were invited for Experiment 1 and for Experiment 2. We have explained this more clearly in the manuscript.

- Lines 318-321 ‘Randomisation with quotas on HL was used to assign women to the first or second experiment. This ensured that approximately the same number of participants with low and high HL would participate in the first and second experiments and be assigned to a presentation format.’

e. How have you excluded that participants of Experiment1 participate in the subsequent Experiment2?

RESPONSE: This was done by the panel. They had the information of the possible participants and selected women based on the questions asked in advance, i.e., the HL questions and questions regarding the exclusion criteria, to participate in the first or second experiment.

- Lines 137-138. ‘Participants in Experiment 1 were excluded from participating in Experiment 2 by the panel.’

f. Which questions about medical knowledge and medical education have been included (reference), where reported, and why at all?

RESPONSE: We thank the editor for this comment. We have now added the questions about medical knowledge and education to the footnote of Table 4. These questions were asked because having medical education and/or medical knowledge can influence the results of the comprehension questions and this can be a particular problem if there are differences between the groups with regards to medical education and/or medical knowledge. We therefore checked whether there were differences in the results of these questions between the groups assigned to the different formats. This turned out not to be the case, as also indicated in the footnote of Table 4.

- Lines 229-231 ‘The survey also included questions about the medical knowledge and medical education of participants to check afterward whether there were any differences between the groups.’
- Lines 334-337 ‘^b^Medical education was assessed using one question asking whether a participant had medical, paramedical, or nursing training. ^c^Medical knowledge was assessed using three questions about medical knowledge in general, knowledge about breast cancer, and knowledge about breast cancer treatments, measured on a 7-point scale (1=no knowledge, 7=a lot of knowledge) [51].’

g. 1 decimal might be sufficient for age M and SD

RESPONSE: We have changed this in the manuscript.

- Lines 233-234 ‘Questions from the first experiment were pre-tested among 67 women between 18-74 years (M=34.9; SD=15.8) without breast cancer.

h. What was the order of the comprehension items?

RESPONSE: The gist comprehension questions were asked before the verbatim comprehension questions. We have now also indicated this in the manuscript.

- Line 231-232 ‘The gist comprehension questions were asked before the verbatim comprehension questions.’

8) Results
a. Figures that illustrate the main effects and (non-)interactions with regard to gist and verbatim comprehensions would be very helpful.

RESPONSE: We have added this information in Supplemental 4.

- Lines 340-342 ‘The ordinal logistic regression results for the interaction and main effects of format and health literacy on comprehension from both Experiments 1 and 2 described below are tabulated in S4.’

b. Figure 4
i. Commas on the y-axis!?
ii. The figure showing sample-based data requires uncertainty intervals.

RESPONSE: We thank the editor for pointing out this error. We have changed the commas into full stops and have added the uncertainty intervals.

c. What is low, middle, high education?

RESPONSE: We agree with the editor that this requires more explanation. We have added this information to the footnote of Table 4.

- Lines 331-334 ‘^a^Low education = primary education or pre-vocational secondary education; middle education = secondary vocational education, senior general secondary education, pre-university education; high education = university of applied sciences or university.’

d. Table 5 does not need a total column

RESPONSE: We have removed the total columns from Tables 5 and 6.

e. Two decimals for test values and confidence intervals might be sufficient (APA)

RESPONSE: We thank the editor for pointing this out. We have adjusted the number of decimal places throughout the Results section in the manuscript and in Supplemental 3.

f. L.463 (you pointed above on Bonferroni adjustments with regard to the large number of secondary outcome analyses), but here risk perception is considered to reveal an effect – please check across the results whether you corrected as planned.

RESPONSE: We have checked this, but we applied the Bonferonni correction with SPSS to all tests for pairwise comparisons.

9) Discussion
a. Why women with low HL did comprehend less about survival rates but not less about side effects?

RESPONSE: This is indeed an interesting point. The information about side-effects was generally less well understood. Perhaps this information was complex for everyone, including those with higher HL. As a result, no difference may have been found between people with higher and lower health literacy. We have added this to the Discussion.

- Lines 458-460 ‘Also regarding the side-effects, the difficulty of the information may have played a role. The information about side effects was generally not well understood, both by people with higher and lower HL.’

10) References
a. See 13 and 45, there are variations across the references in capitalisation and abbreviations of journals, please ensure references consistency!

RESPONSE: There were indeed differences in the use of abbreviations and capitalization. We have adjusted this. However, references 13 and 45 concern two different journals, namely the journal *BMC Medical Informatics and Decision Making* and the journal *Medical Decision Making*.
