# Supplementary material for: Presenting decision-relevant numerical information to Dutch women aged 50–70 with varying levels of health literacy: Case example of adjuvant systemic therapy for breast cancer
Source: PLoS One. 2024 Sep 3;19(9):e0309668. doi: 10.1371/journal.pone.0309668 (PMC11371237; doi:10.1371/journal.pone.0309668)
Supplement: Attachment — Submitted filename: Response to Reviewers July 2024.docx [file pone.0309668.s007.docx]

Dear Editor,

Thank you for the comments and the opportunity to address these points in our manuscript entitled *‘Presenting decision-relevant numerical information to Dutch women aged 50-70 with varying levels of health literacy: case example of adjuvant systemic therapy for breast cancer*’ (PONE-D-23-32983R2). We have responded to the outstanding points as described below. Changes to the manuscript are underlined in our responses and indicated by tracked changes in the revised manuscript.

Additional Editor Comments:

Dear Authors,
You have extensively addressed concerns and corrected where necessary. Thank you! Finally, one thing remains, which centers around low-literacy findings/non-findings of Experiment 2.
In your preregistration you hypothesised:
H2a. People provided with information in a visualization [..] will report more adequate comprehension of the trade-off between survival rates and likelihood of side-effects (gist comprehension trade-off) compared to people provided with the information in numbers only (condition 2a and 3a). Health literacy will moderate this relation, in the sense that those with lower/inadequate health literacy will be better supported in comprehension of the trade-off with the visualization (condition 2b – condition 3b) compared to numbers only (condition 2a and 3a) compared to those with higher/adequate health literacy. Similarly, H2b.
Then you tested that according to the manuscript:
"in both experiments the analyses with comprehension as outcome were performed with cumulative odds ordinal logistic regression with proportional odds (instead of ANOVAs)."
The power calcuation, however, was made for an ANOVA. And you in the discussion: "However, it should be noted that the expected interaction-effects were ordinal-interactions rather than full crossover interactions, therefore the statistical power to detect the expected interactions is lower than the a priori calculated 90% and 91%."
Now, Experiment 2 that varies the presentation of probability came with cell samples between 17 and 26 participants. You referred to both the power calculation (which was made under different assumptions) and limitation section in your discussion as cited.
Now we can say that Experiment2-Comprehension was less likely to detect any difference among the factor stages (than planned), but even less likely to enable planned comparisons as you hypothesised them in the preregistration.
Btw: With which tool have you done the power calculation?
On the other side you wrote about applying Bonferroni correction but your results seem to be interpreted still consistently under alpha<.05. How do I recognise your adjustment? For the example: “Regarding ‘feeling informed’ (H3), we found an interaction between HL and format, F(4, 274) = 2.67, p = .032, partial η2 = .04.“ If alpha is adjusted, this may typically would not count as being significant. Note that according to the preregistration the interaction on „feeling informed“ is exploratory.

Taken together, please check how both low power and multiple testing correspond with your results and discussion on the interaction with HL; according to my understanding the manuscript would benefit if you make more concrete that the question whether low HL people comprehended your interventions differently than high HL people could not be reliably addressed by your studies.

RESPONSE: We thank the editor for his comments regarding low power and multiple testing in the second experiment. We agree with the editor that our study has limitations in answering the question of whether people with low health literacy were better supported in understanding the side-effects when visualizations were used than people with high health literacy. We have now stated this more clearly in the Discussion section.

As mentioned in the pre-registration, we used the software programs G*Power and PASS for the power calculations. We have now also added this to the manuscript in the Methods section.

Regarding the Bonferroni correction, this adjustment has been incorporated into simple main effects results. We have now also indicated this in the manuscript in the Results section. We hope that this makes it more recognizable in the manuscript.

- Lines 293-294 ‘An a priori power analysis was performed based on a 3x2 (Experiment 1) and a 5x2 factorial ANOVA design (Experiment 2) with interaction-effect using the software programs G-power and PASS.’
- Lines 383-385 ‘For format C (visualised probability information without description), there was a difference in the average score on feeling informed between women with low and high HL, F (1,272) = 7.75, p = .006 after Bonferroni correction, partial η^2^ = .03.’
- Lines 535-537 ‘This implies that the question of whether people with lower health literacy levels would benefit more from the formats with the visualizations than people with higher health literacy could not be answered reliably.’
